# Lignocellulose Extraction from Sisal Fiber and Its Use in Green Emulsions: A Novel Method

**DOI:** 10.3390/polym14112299

**Published:** 2022-06-05

**Authors:** Sippi Pirah, Xiaodong Wang, Muhammad Javed, Keenjhar Simair, Bijia Wang, Xiaofeng Sui, Changrui Lu

**Affiliations:** 1 Key Laboratory of Science and Technology of Eco-Textile, Ministry of Education, College of Chemistry, Chemical Engineering and Biotechnology, Donghua University, Shanghai 201620, China; wxd_19961107@163.com (X.W.); javed@mail.dhu.edu.cn (M.J.); bwang@dhu.edu.cn (B.W.); suixf@dhu.edu.cn (X.S.); 2 Department of Chemistry, GC University, Hyderabad 71000, Pakistan; keenjharsimair@gmail.com

**Keywords:** phosphoric acid, sisal suspension, oil in water emulsion, Pickering emulsions

## Abstract

Regenerated lignocellulose nanofibrils (RLCNFs) have recently piqued the interest of researchers due to their widespread availability and ease of extraction. After dewaxing, we treated sisal fiber with alkali, followed by heating and agitation, to obtain RLCNFs, which were then vacuum oven-dried. We used a variety of characterization techniques, including XRD, SEM, and FT-IR, to assess the effects of the alkali treatment on the sisal fiber. Various characterizations demonstrate that lignocellulose fibrils have been successfully regenerated and contaminants have been removed. In addition, employing the RLCNFs as a stabilizer, stable Pickering emulsions were created. The effects of RLCNF concentration in the aqueous phase and water-to-oil volume ratio on stability were studied. The RLCNFs that have been produced show promise as a stabilizer in Pickering emulsions.

## 1. Introduction

Biomass is one of the cost-effective, eco-friendly, and readily available resources around the world. Biomass resources contain cellulose, lignin, extractives, and traces of hemicellulose. They include crop residues such as corn straw, wheat straw, rice husk [1], sugarcane bagasse [2], pineapple peel [3], oil palm fruit [4], bamboo chips [5], and sisal fibers [6]. Among them, sisal fiber biomass comprises relatively high cellulose content (64.4–65%), low hemicellulose (20–27%), and a small amount of lignin (9.7–13%) [7]. Moreover, sisal fibers are easily available (2% of the world’s plant cultivation). They have low density and are mechanically strong, biocompatible, and biodegradable. Each sisal plant (*Agave sisalana)* produces 200–250 leaves during its 10-year life span, and one dry leaf holds 4% of fiber by mass [8]. Due to all the aforementioned properties, sisal fibers have been used to produce cellulose nanowhiskers [6], surgical sutures [9], textile materials [10], composites [8], Pickering emulsions [11], and much more.

Nanocellulose denotes cellulose that is in nanometer size at least in one dimension. The three types of nanocellulose are cellulose nanofibrils (CNFs), cellulose nanocrystals (CNCs) [12], and bacterial nanocellulose (BNC) [3]. The extraction of nanocellulose from wood biomass needs alkali treatment, bleaching, and other harsh methods that not only raise environmental concerns but also render the overall process expensive. Moreover, the hydrophilic nature of cellulose due to its hydroxyl groups makes it unsuitable for a variety of applications, such as Pickering emulsion stabilizers, hydrophobic films, and composites [13]. In nature, lignin is strongly bonded to cellulose with van der Waals forces and hydrogen bonding, providing rigidity and hydrophobicity to cellulose [14]. Cellulose, lignin, and hemicelluloses interlink with each other, forming a polymer network. The strong intermolecular forces among them strongly resist dissolution in a common solvent [15]. To overcome this problem, regenerated lignocellulose nanofibrils (RLCNFs) have gained a huge research interest recently due to their availability and comparatively easy extraction. Recent research shows that residual lignin in CNFs enhances their properties, specifically hydrophobicity, mechanical stability, thermal stability, and antioxidant activity [15,16,17,18]. To date, various methods have been reported to extract lignocellulosic nanofibrils (LCNFs), including chemical methods (such as pulping, inorganic acid hydrolysis [19], enzymatic hydrolysis [20], and TEMPO oxidation [21]), physical treatments (such as high-pressure homogenization, ultra-sonication, and grinding [22]), their combination [23], or organic acid hydrolysis [17]. Jia et al. used phosphoric acid to obtain regenerated cellulose [24], whereas Zhang et al. used a simple glycerol swelling technique along with N-methyl morpholine-N-oxide monohydrate solvent to dissolve lignocellulose. Lignocellulosic crystals were obtained from sugarcane straw by Billato et al. [25] and from bamboo chips by Lu et al. [5].

Surfactants and hydrocolloids play an important role in our daily lives, from the household level to the industrial level. They are being used in food, pharmaceutics, paints, dyes, and the cosmetic industry. Owing to their large-scale use, industries are seeking bio-based and eco-friendly alternatives that can reduce their carbon footprint [26]. The global surfactant market is worth USD 19 billion per year, just for detergents, and a massive amount of surfactants are released into the environment that may or may not be degraded [27]. Therefore, the development of green emulsifiers has gained huge attention recently. Pickering emulsification is a strong candidate in this context due to its practicability in various applications.

Ramsden [28] and Pickering [29] pioneered the Pickering emulsions that are stabilized by solid particles at the fluid–fluid interface, making them preferable to traditional emulsions [30]. Inorganic molecules or hydrocarbons are of great interest in conventional emulsions, but their use in the pharmaceutical and food industries is largely limited due to low biodegradability and biocompatibility [31]. Lignocellulosic nanocrystals or fibers are reported to be suitable for emulsification over other natural emulsifiers [32]. Chen et al. obtained lignocellulosic nanocrystals from pineapple peel and used them as Pickering emulsion stabilizers and reported that residual lignin enhanced the stability of Pickering emulsion [3]. Notably, we could not find any report in the literature where sisal fiber was utilized to prepare RLCNFs, and their role as an emulsifier was completely demonstrated.

In this study, we developed a novel combinatorial strategy to prepare RLCNFs directly from sisal fiber in a two-step process that can easily be scaled up. The major step involves the dissolution of fiber and ultra-sonication to produce an oil-in-water emulsion. The fibers were simply cut into small pieces before being soaked in water. Heat and agitation were used to dissolve them in phosphoric acid. The dissolving conditions, fiber, water, and phosphoric acid ratios were optimized (1:5:50 at 60 °C for 5 h). Water was poured into the produced solution to regenerate the lignocellulosic nanofibers until pH 7 was reached. The dissolved fibers settled, and we removed the top layer of clear water to obtain a more concentrated solution. Finally, we used centrifugation to obtain a highly concentrated solution with a solid content of 5.0% and further characterized it by Fourier transform infrared spectroscopy (FT-IR), X-ray diffraction (XRD), transmission electron microscope (TEM), and optical microscope. Finally, to make a Pickering emulsion using n-hexadecane, we employed RLCNFs as the water phase. There are other kinds of paraffin, such as n-octadecane, in addition to n-hexadecane. The particle size of the resultant emulsion was observed via optical microscopy.

## 2. Experimental Section

### 2.1. Materials

Sisal fiber was purchased from Guangxi Sisal Group Co. Ltd., one of the leading sisal manufacturers in China. Phosphoric acid was purchased from Shanghai Titan Technology Co. Ltd., Shanghai, China, and n-hexadecane was obtained from Sinopharm Chemical Reagent Co. Ltd., Shanghai, China. Deionized water was supplied by Laboratory Water Purification System, Hitech Instrument Co. Ltd., Shanghai, China.

### 2.2. Preparation of RLCs

The sisal fiber was cut into 5 mm pieces with scissors before being crushed in a shredder (2 min). After grinding, 85% phosphoric acid was used, and 5 g of sisal hemp in a mass ratio of 2:1 was placed in beakers and manually mixed for 5 min. Stirring at 450 rpm was performed for 8 h at 35 °C, or until sisal fibers were completely dissolved. Dissolved sisal hemp was rehydrated with significant amounts of water until pH = 6–7, and solid content was determined (7–12%).

### 2.3. Preparation Pickering Emulsions

Firstly, RLCNF solution samples with solid contents of 0.5 wt.%, 0.3 wt.%, and 0.1 wt.% were prepared. Secondly, three different oil/water emulsions with ratios 1:9, 3:7, and 5:5 were prepared and homogenized with a homogenizer for 3 min to make a uniform emulsion. The oil phase includes n-hexadecane and n-octadecane. The prepared emulsions were kept at room temperature for 1 h, 1 day, 5 days, 7 days, and 20 days to test their stability at 50 °C.

### 2.4. Characterization

An electronic balance (ME204E, Mettler Toledo, Columbia, MD, USA, accuracy 0.1 mg) was used to measure the weight of samples. The temperature of the solution was detected by a thermocouple (DT-8891E, CEM, Macau, China) during the dissolution process. IKA Overhead Stirrer EUROSTAR 60 control (IKA, Munich, Germany) was used for stirring the solution. The morphology of prepared raw sisal and RLCNFs was observed using an optical microscope (E100, Nikon, Tokyo, Japan), field emission scanning electron microscope (FE-SEM) (S-4800, Hitachi, Tokyo, Japan), cryo-scanning electron microscope (Cryo-SEM) (EVO-MA10, ZEISS, Jena, Germany), and transmission electron microscope (TEM) (JEM-2100, JEOL, Tokyo, Japan). A homogenizer (T18, IKA, Munich, Germany) was used in the process of emulsion homogenization. Dynamic light scattering (DLS) (Nano-ZS, Malvern, UK) and a laser particle size analyzer (S3500, Microtrac, Montgomeryville, PA, USA) were used to examine the particle size distribution of LNPs and emulsions at room temperature, respectively.

## 3. Results and Discussion

### 3.1. Morphology of Raw Sisal Fibers

**Figure 1:**
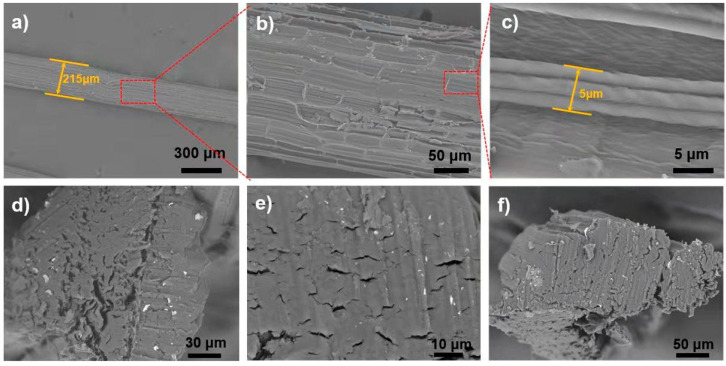
Scanning electron micrograph of different segments of raw sisal fiber: (**a**) Cross-sectional view of raw sisal fiber and visual demonstration of numerous microfibrils intertwined with each other. (**b**) SEM image of the red rectangular area selected in panel (**a**). (**c**) Magnified SEM view of the marked red rectangular area from panel (**b**). (**d**–**f**) Cross-sections of a raw sample with different magnifications.

**Figure 2:**
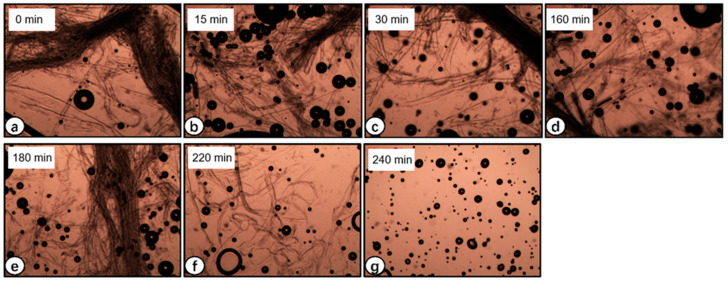
Optical micrographs of sisal fibers samples hydrolyzed in H_3_PO_4_: (**a**) 0 min; (**b**) 15 min; (**c**) 30 min; (**d**) 160 min; (**e**) 180 min; (**f**) 220 min; (**g**) 240 min.

The SEM images of raw fiber are critical for examining the structure and alignment of the fiber‘s microscopic units of microfibrils. The pattern of the microfibrils, once aligned and compacted, requires a stronger pre-breakdown (mechanical or chemical) and additional acid treatment to disintegrate them. This breakdown of internal forces of microfibrils results in a fully homogeneous solution. Raw sisal fiber is composed of numerous microfibrils, as shown in SEM images in Figure 1. Microfibrils have a diameter of 10–15 μm, whereas raw sisal fibers have a diameter of 200–500 μm (Figure 1b). A magnified view of chosen microfibrils from a portion of panel b is shown in Figure 1c. The original sisal fiber‘s constituent microfibrils are compactly aligned along the main axis, and non-fibrous components can be seen on the surface (Figure 1d). The morphology of raw sisal fibers that we observed is consistent with earlier research studies [33]. This nanofibrillar assembly disintegrates into a homogeneous viscous solution after acid hydrolysis and continues stirring for 4 h (Figure 2g).

### 3.2. Optical Microscopy of Raw Sisal Fiber Dissolution

The treatment included 85% phosphoric acid with continuous stirring to overcome the cohesive forces between the lignocellulosic fibers. Figure 2a–g depicts the optical micrographs of the samples taken between 0 and 4 h, respectively. It can be clearly seen that the treatment effectively caused the dissolution of sisal fibers, causing a gradual increase in viscosity with time. The increase in viscosity is proof of the uniform dispersion of lignocellulosic nanofibers (LCNFs) in the suspension. Our findings are in agreement with Trifol et al. [34]. By using the method, they used to extract nanocellulose from sisal fibers, they observed that the sisal fibers gradually became more separated and that the sample eventually reached the rheological percolation threshold. The cellulose fibers are entirely dissolved in the reagent after four hours of treatment, as shown in Figure 2g.

Lignocellulose in sisal fibers, being highly crystalline and insoluble in water and possessing strong intra- and intermolecular hydrogen bonds [7], is not a good candidate for emulsion stabilizers. Phosphoric acid disrupts these intermolecular forces, and the resultant regenerated lignocellulosic nanofibers are promising materials for gelling and emulsion stabilizers [24]. The dissolution of lignocellulosic nanofibers is the outcome of an esterification reaction due to phosphoric acid that is reversed during the regeneration of RLCs with water [24]. Phosphoric acid has been reported to dissolve cellulose and form stable dispersions of cotton and sisal fibers [35], which in our case were effectively dissolved in the same way, as evidenced by the optical micrographs.

### 3.3. Morphology of RLCs

**Figure 3:**
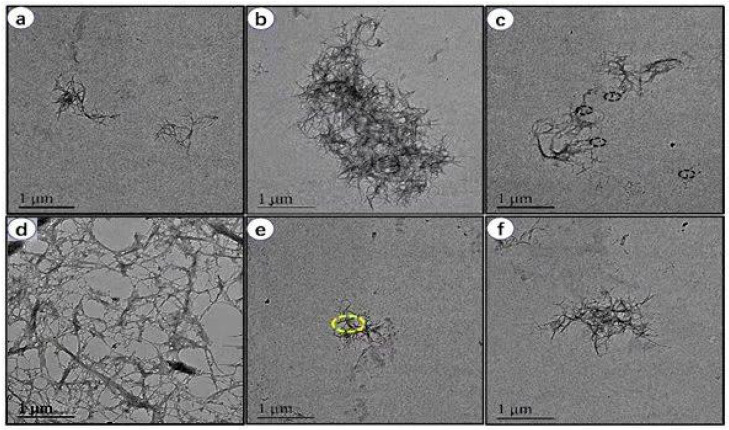
TEM images of lignocellulosic nanofibers (LCNFs): (**a**) TEM image of raw sisal fiber without phosphoric acid treatment. (**b**–**e**) TEM micrograph of nanofiber after treatment with phosphoric acid. The size of nanofiber is reduced significantly from micrometer to nanometer range. (**f**) Magnified view of selected yellow circle area from panel (**e**).

The regenerated lignocellulose nanofibers and suspension liquids were seen using TEM. The micrographs in Figure 3a–f were taken at a magnification of 1 μm. The TEM image of sisal fiber without phosphoric acid treatment is shown in Figure 3a. As seen in Figure 3b–e, sisal fiber diminishes in size when treated with phosphoric acid. Finally, as demonstrated in Figure 3f, the size of the nanofiber decreases to the nanoscale. It is evident in the micrographs that phosphoric acid treatment changed the morphology of raw sisal fibers and the resultant fibers were in nanoscale diameter. Moreover, the nanofibers were more entangled and contained nanometric globular particles attached to the surface, as seen by the yellow dotted line in Figure 3e. These adhered globules were attributed to lignin residues, and this is the typical morphological feature of lignocellulosic nanofibers reported previously by Guo et al. [36].

### 3.4. FT-IR Analysis

**Figure 4:**
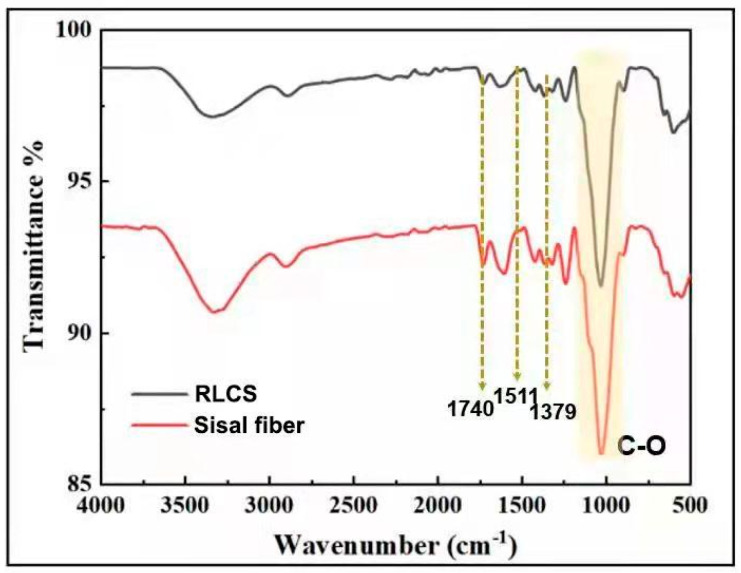
FT−IR spectra of raw sisal fibers and RLCs.

The FT-IR spectra of raw sisal fiber and RLCs are shown in Figure 4. Stretching vibrations of CH and OH are responsible for the main peaks between 3400 cm^−1^ and 400 cm^−1^. Identical stretching in spectra demonstrates that no chemical deterioration but only intermolecular changes happened during regeneration of sisal fibers. The discrete peak at 1740 cm^−1^ is ascribed to uronic ester and/or acetyl groups of hemicelluloses, or the carboxylic group of ferulic and p-coumaric acids from lignin or hemicellulose. Less stretching obtained in this region for RLCs indicates the removal of some of these mentioned groups during the washing process [5,37,38]. The peak at 1511 cm^−1^ corresponds to the aromatic ring of lignin’s C=C stretching vibration, whereas the C–H asymmetric distortions are seen at 1379 cm^−1^, and C–O stretching is visible in the 950–1200 cm^−1^ area [5]. These characteristic stretching peaks in spectra indicate that these key functional groups are present in both raw sisal and RLCs. However, all the above peaks are in good agreement with the previous reports.

### 3.5. XRD

**Figure 5:**
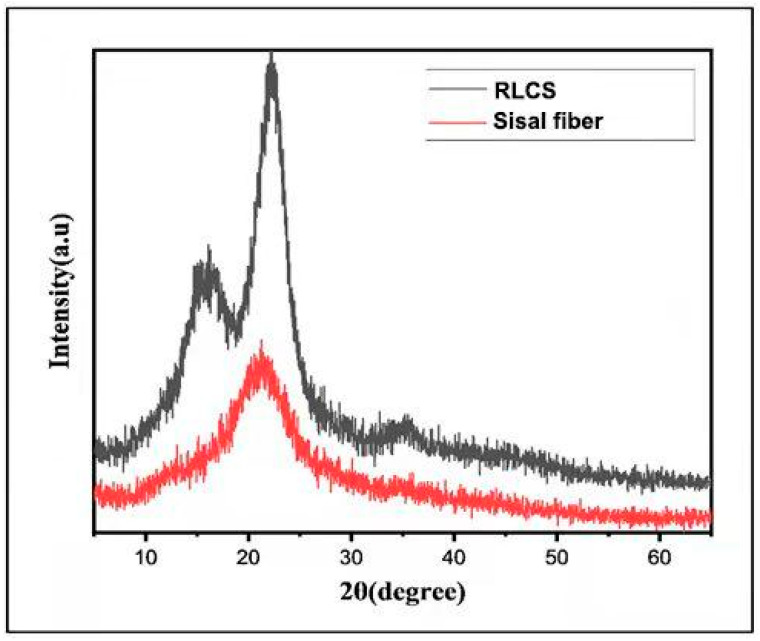
XRD analysis of raw sisal fibers and RLCs.

Figure 5 represents the XRD spectrogram of raw and regenerated sisal fibers. The pattern elucidates the presence of semi-crystalline amorphous peaks of typical cellulose I as reported in the literature [5,39]. Many pretreatment processes (such as ball milling) reduce the cellulose content of lignocellulosic biomass, rendering it less suited for a variety of applications [2]. The characteristic peaks of 2θ angles at around 15°, 22.5°, and 34.5° are depicted by (1–10) and (110) planes, (200) planes, and (004) planes respectively. The peak showing crystal plane (I_200_) at 2θ is obtained at around 22.5° for both raw and regenerated sisal fibers, whereas the amorphous contribution is at 18°, which is more obvious in raw sisal fibers [25].

The degree of crystallinity is a characteristic feature of nanocellulose that regulates its physical, chemical, and mechanical properties in a manner conducive to spatial arrangement and symmetry. Based on biomass and pretreatment methods used, the crystallinity index of cellulose nanocrystals ranges from 70 to 88% [2,20,25]. It is reported that acid hydrolysis increases the crystallinity index by removing some of the amorphous sections of cellulose material [5], which in this case is phosphoric acid. Our findings are in agreement with the previous reports where acid hydrolysis resulted in the improved crystallinity of lignocellulosic nanofibrils [5,25].

### 3.6. Stabilization of Pickering Emulsion

**Figure 6:**
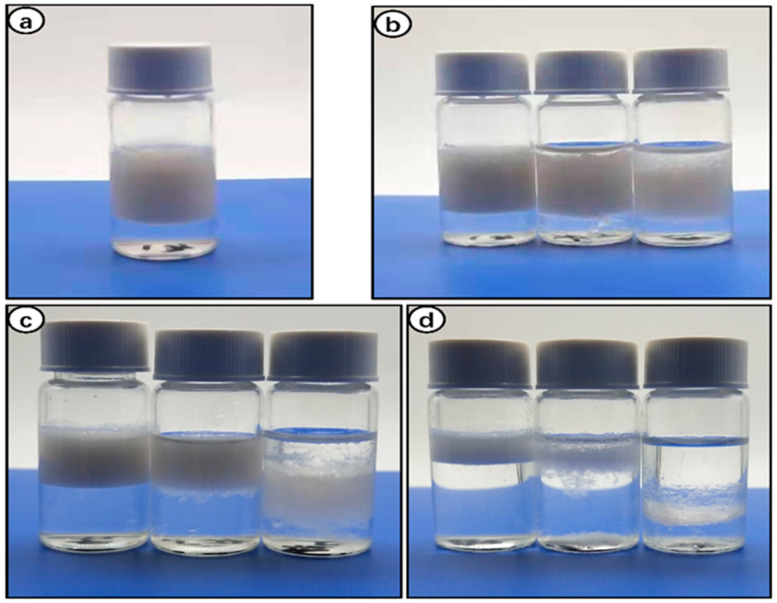
Oil/water Pickering emulsions after dispersing at 10,000 rpm for 3 min: (**a**) O/W, 1:9; RLCs = 0.5% (oil phase = n-hexadecane, water phase = RLC suspension). (**b**–**d**) From left to right, O/W ratio = 1:9, 3:7, 5:5. (**b**) RLCs = 0.5%; (**c**) RLCs = 0.3%; (**d**) RLCs = 0.1%.

Pickering emulsion formation and stability depend on various factors, including the oil phase, particle concentration, and the ratio of dispersed and continuous phases [40]. Hexane is one of the more hydrophobic organic solvents, making it suitable for stable Pickering emulsions. Liu et al. reported that hydrophilic organic solvents form a blurred oil–water interface, leading to poor adsorption of CNWs [11]. Figure 6 shows the digital photographs of Pickering emulsions stabilized using n-hexane as the oil phase and three concentrations (0.1%, 0.3%, and 0.5%) of RLC suspension as the water phase, and both phases were mixed with three different oil/water ratios (1:9, 3:7, and 5:5). It is evident that the most stable emulsification was obtained in 0.5% RLCs mixed with hexane with an O/W ratio of 1:9 (Figure 6a). The integration of RLCs obtained through solubilization and regeneration in biphasic (O/W) systems caused the formation of stable emulsion because of the development of an interfacial barrier by adsorption at the oil–water interface [26,36]. Moreover, the emulsion ratio increased as the concentration of RLCs was increased from 0.1 to 0.5%. Our results coincide with the previous reports where increasing concentrations of CNWs [11], LCNFs [36], and 0.5% CNFs [4] enhanced the emulsification ratio.

**Figure 7:**
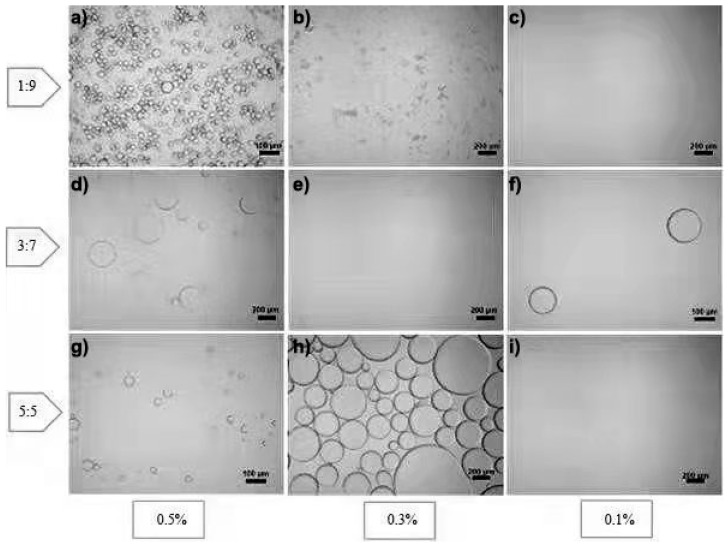
Optical micrographs of the Pickering emulsions of hexane in RLC suspension. Optical micrographs show that (**a**–**c**) the O/W ratio is 1:9, the oil phase is n-hexadecane, and the water phase is sisal suspensions, where the contents of sisal suspensions were 0.5%, 0.3%, and 0.1%, respectively. (**d**–**f**) Optical micrographs show that the O/W ratio is 3:7, the oil phase is n-hexadecane, and the water phase is sisal suspensions, where the contents of sisal suspensions were fixed to 0.5%, 0.3%, and 0.1%, respectively. (**g**–**i**) Optical micrographs show that the O/W ratio is 5:5, the oil phase is n-hexadecane, and the water phase is sisal suspensions, where the contents of sisal suspensions were 0.5%, 0.3%, and 0.1%, respectively.

The O/W ratio of 1:9 in all concentrations of RLCs formed a stable emulsion without flocculation or sedimentation (Figure 6b–d). Conversely, the surface tension of the interface was lowered because the hexane–water junction was not twisted, but all concentrations of RLCs at a ratio of 5:5 showed poor emulsification performance (Figure 6d). The most probable justification for this observation is that an increase in the ratio of the dispersion phase results in phase inversion and separation [11]. Figure 7 depicts the optical micrographs of the respective emulsions, and it can be clearly seen that the decrease in O/W ratio is strongly associated with a decrease in droplet size and narrow size distribution.

When RLC concentration was increased to 0.5%, the average droplet size obtained was 37 μm in an O/W ratio of 1:9. Furthermore, the size distribution of the droplets was also uniform compared to 0.3% and 0.1%. It corresponds to the role of lignocelluloses in emulsification. We can see from Figure 6b that at lower concentrations the emulsion system was not stabilized, leading to coalescence.

The coalescence can be attributed to the weak interfacial barrier posed by large-sized droplets [41,42]. Previous studies reported by Guo et al. on LCNFs [36] and Chen et al. on LCNCs [3] correlate with our finding that the smaller droplet size favored the stability of Pickering emulsions. They justified it with the fact that the increased concentration of lignocellulosic nanofibers enabled the access of more fibrils to droplets, resulting in the stable formation of stable emulsions. The droplet diameter (Figure 7e,f,h,i) for 0.3% and 0.1% RLC concentrations is greater than 100 μm, and the evidence is supported by digital photographs of emulsions of corresponding concentrations (Figure 6c,d). Xia Li et al. demonstrated that 0.8% concentration was the minimal concentration to promote emulsification in cellulose nanofibers extracted [4]. Lignocellulosic residues in our RLCs may tend to enhance emulsification according to previous reports [3,32,36]. Cellulose nanocrystals alone need a higher concentration to form stable Pickering emulsions, while lignocellulose, being hydrophobic, augments the emulsification properties of cellulose by promoting the oil–water interface barrier [43].

## 4. Conclusions

For the first time, we demonstrated that dissolving sisal fibers with phosphoric acid, a green solvent, has rapid and efficient non-derivative dissolution; is a benign, non-volatile, cost-effective, recyclable green solution; and may generate excellent results. After being dissolved in phosphoric acid and regenerated in water, sisal fiber can be used as an excellent emulsifier for stabilizing Pickering emulsions. Furthermore, using RLCNFs as a stabilizer resulted in stable Pickering emulsions with a moderate rate of settlement. They can be utilized for dust management, tack coating, fog sealing, and mixing fine gradations. Many plant-derived compounds are water-insoluble; however, they can be employed as insoluble particles with sizes ranging from nanometers to micrometers that can stabilize oil–water interfaces. RLCNFs create viscous aqueous suspensions and solutions, which limit the mobility of oil droplets and increase the stability of emulsions. Because of their qualities, lignocellulosic stabilizers are particularly well suited to technical emulsions, such as those formed from crude oil, as they can reduce the viscosity of the emulsion. In the future, their use is anticipated in medical, food, cosmetics, and other industries. After stabilizing paraffin for 8 months, the emulsifier demonstrated good stability.

## Data Availability

Data is contained within the article.
